# Diagnostic Challenge of Appendiceal Endometriosis: A Case Report

**DOI:** 10.7759/cureus.96376

**Published:** 2025-11-08

**Authors:** Andreia Rodrigues, Rui Sousa, Inês Morujão, Oliveira Martins

**Affiliations:** 1 General Surgery, Unidade Local de Saúde de São José, Lisboa, PRT

**Keywords:** abdominal pain, acute appendicitis, appendiceal endometriosis, endometriosis, laparoscopy

## Abstract

Endometriosis is a chronic, estrogen-dependent disease affecting women of reproductive age. Although extragenital involvement is not uncommon, appendiceal endometriosis is a rare entity that may present with acute abdominal pain, often mimicking acute appendicitis and posing a diagnostic challenge. We report the case of a 28-year-old woman who presented with right lower quadrant abdominal pain and clinical suspicion of acute appendicitis. She underwent a laparoscopic appendectomy. Histopathological examination of the specimen revealed endometriosis of the appendix. The postoperative course was uneventful. This case highlights the diagnostic overlap between appendiceal endometriosis and acute appendicitis, in line with previous reports. Consistent with earlier findings, appendiceal involvement may occur independently or in association with other pelvic sites. The absence of luminal inflammation, as observed here, differentiates this case from those with concurrent inflammatory appendicitis. Routine histopathologic assessment remains crucial, as many lesions are microscopic and clinically silent. Laparoscopy plays a pivotal role by allowing direct visualization, safe resection, and evaluation for additional endometriotic implants. Appendiceal endometriosis should be considered in women presenting with acute or recurrent right lower quadrant pain. Laparoscopy combined with systematic histological examination provides definitive diagnosis and optimal management, helping prevent misdiagnosis of this rare but clinically significant entity.

## Introduction

Endometriosis is a chronic, estrogen-dependent condition defined by the presence of endometrial glands and stroma outside the uterine cavity [1,2]. It affects approximately 6-10% of women of reproductive age and is often associated with pelvic pain, dysmenorrhea, dyspareunia, and infertility [1,3,4]. Symptoms are typically related to the location of the lesions, with the pelvis being the most commonly affected site. However, extragenital involvement, particularly of the gastrointestinal tract, can occur in up to 37% of cases [3,4].

Appendiceal endometriosis is a rare manifestation, reported in 0.05-1.69% of patients with endometriosis and more frequently among those undergoing surgery for deep infiltrating endometriosis [1,3,5]. Clinically, it can mimic acute appendicitis, making diagnosis challenging, especially in women presenting with right lower quadrant abdominal pain [4,6].

Given this overlap, appendiceal endometriosis should be considered in the differential diagnosis of acute abdomen in women of childbearing age. In this article, we present a clinical case of appendiceal endometriosis to illustrate its diagnostic complexity and surgical relevance.

## Case presentation

We present the case of a 28-year-old woman with no significant past medical history, no regular medications, and no known drug allergies. She had one child and was otherwise healthy. Her initial presentation to the emergency department in June of 2024 was due to dysuria and lower abdominal discomfort, and she was discharged with a presumptive diagnosis of urinary tract infection after empirical treatment.

Four days later, she returned to the emergency department with persistent pain localized to the right iliac fossa. On physical examination, she exhibited localized tenderness in the right lower quadrant with an equivocal peritoneal reaction. Her vital signs were stable. Laboratory investigations were notable for a significantly elevated C-reactive protein level of 107.7 mg/dL, while her white blood cell count was within normal limits (Table 1).

**Table 1:** Complete laboratory findings on the day of the intervention

Test	Result	Reference range
Complete blood count		
Red blood cells	4.57 × 10^12^/L	3.8–5.0
Hemoglobin	13.8 g/dL	12.0–15.0
Hematocrit	40.4%	35–46
Mean corpuscular volume (MCV)	88.4 fL	78.0–96.0
Mean corpuscular hemoglobin (MCH)	30.2 pg	26.0–33.0
Mean corpuscular hemoglobin concentration (MCHC)	34.2 g/dL	31.0–36.0
Red cell distribution width (RDW)	12.1%	11.5–15.5
White blood cells	9.55 × 10^9^/L	4.5–11.0
Neutrophils %	76.5%	40–75
Eosinophils %	0.7%	0.0–6.0
Basophils %	0.3%	0.0–1.0
Lymphocytes %	16.8%	15–45
Monocytes %	5.7%	2.0–11.0
Neutrophils #	7.31 × 10^9^/L	2.0–8.5
Eosinophils #	0.07 × 10^9^/L	0.0–0.6
Basophils #	0.03 × 10^9^/L	0.0–0.1
Lymphocytes #	1.60 × 10^9^/L	0.9–3.5
Monocytes #	0.54 × 10^9^/L	0.2–1.0
Platelets	289 × 10^9^/L	150–450
Coagulation profile		
Prothrombin time (PT) – seconds	13.8 s	9.4–12.5
PT – percentage	80%	70.0–130.0
INR	1.15	0.80–1.20
Activated partial thromboplastin time (aPTT) – seconds	29.6 s	25.1–36.5
aPTT – ratio	1.00	0.8–1.2
Biochemistry		
Glucose	87 mg/dL	60–100
Urea	17 mg/dL	16.6–48.5
Creatinine	0.83 mg/dL	0.51–0.95
Electrolytes		
Sodium	138 mEq/L	136–145
Potassium	4.5 mEq/L	3.50–5.10
Chloride	102 mEq/L	98.0–107
Calcium	9.8 mg/dL	8.60–10.0
Inflammatory marker		
C-reactive protein (CRP)	107.7 mg/L	<5.0

**Figure 1:**
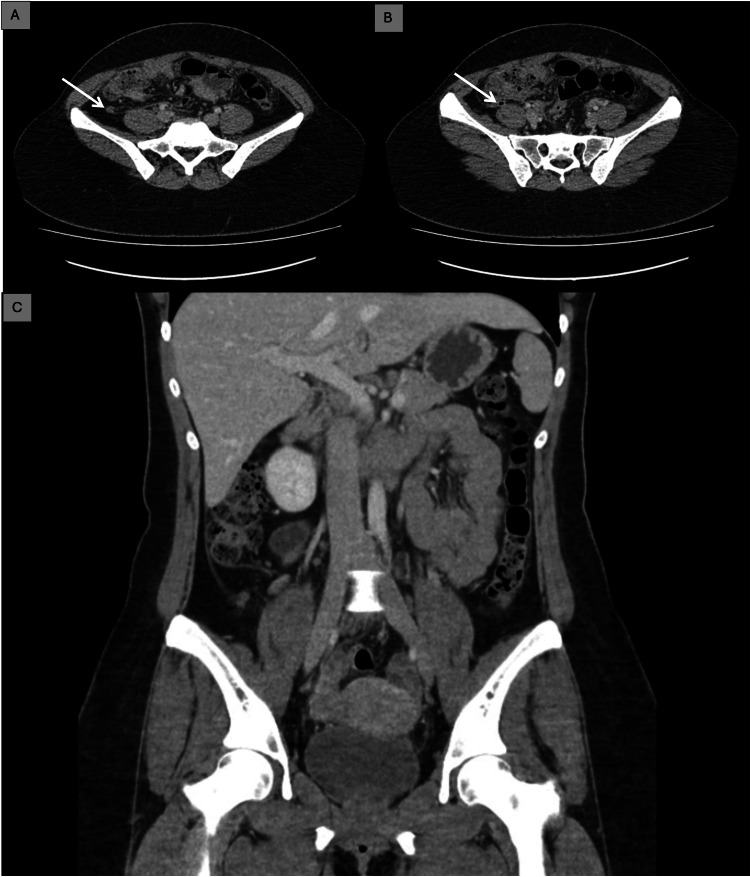
Abdominopelvic CT scan performed in the emergency department. (A, B) Axial views showing findings consistent with appendiceal wall thickening and periappendiceal fat stranding. (C) Coronal view of the pelvic floor without significant findings in the present technique. CT: computed tomography; white arrow: appendix

Given the persistence of symptoms and the inconclusive clinical findings, an abdominal ultrasound was performed, which did not reveal any abnormalities. Due to ongoing pain and the elevated inflammatory markers, an abdominopelvic computed tomography (CT) scan was requested. The CT scan demonstrated imaging features consistent with uncomplicated acute appendicitis, characterized by wall thickening and periappendiceal fat stranding, without evidence of perforation or abscess formation (Figure 1a-1c).

A decision was made to proceed with surgical management. The patient underwent a laparoscopic appendectomy under general anesthesia. Intraoperative exploration revealed a phlegmonous ileocecal appendix without evidence of perforation or periappendiceal collection. Additional findings included marked hyperemia of the pelvic cavity and the presence of multiple adhesions involving adjacent peritoneal surfaces. The procedure was completed without complications.

The postoperative course was uneventful. The patient tolerated oral intake, mobilized early, and was discharged in good condition on the second day post-op. 

**Figure 2:**
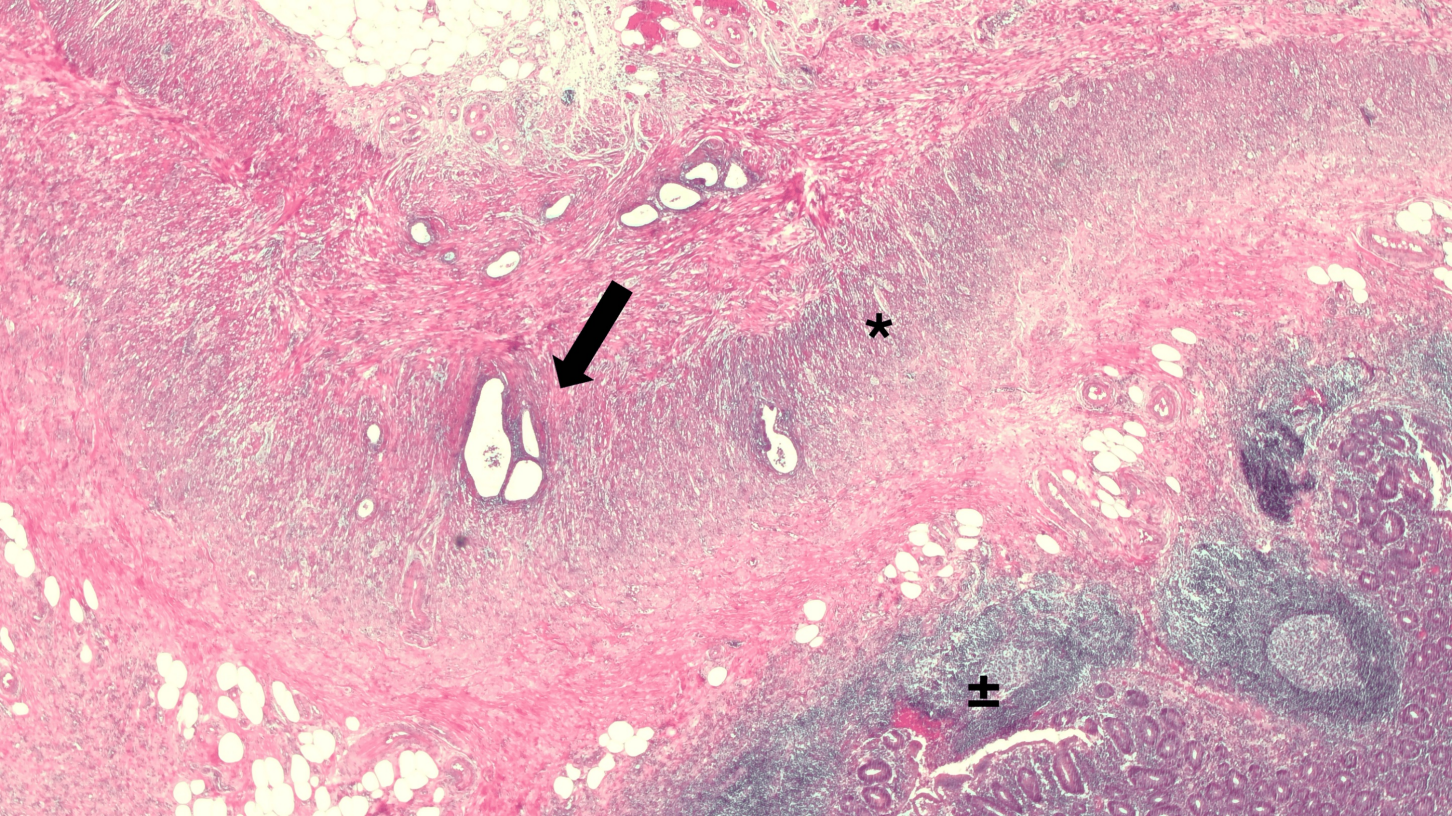
Histopathological image of the resected appendix showing endometriosis Histopathological examination of the resected specimen confirming the diagnosis of appendiceal endometriosis, revealing an ileocecal appendix with an extrinsic inflammatory process and foci of endometriosis within the muscularis propria. ->: foci of endometriosis; *: muscularis propria of the appendix; ±: mucosa of the appendix

Histopathological examination of the resected specimen revealed the diagnosis of appendiceal endometriosis, presenting an ileocecal appendix with an extrinsic inflammatory process and foci of endometriosis within the proper muscular layer, without evidence of an endoluminal inflammatory process (Figure 2).

This finding provided a definitive explanation for the atypical clinical presentation and the intraoperative inflammatory changes. The patient was referred to the gynecology and obstetrics outpatient clinic, where she was prescribed dienogest 2 mg.

## Discussion

Endometriosis involving the appendix is a rare but clinically significant manifestation of extragenital disease. The reported prevalence varies depending on the study population and histopathological criteria applied. Guo et al. [1] found appendiceal involvement in 35.8% of women with stage IV endometriosis, highlighting the importance of careful intraoperative inspection of the appendix. Allahqoli et al. [5], in a comprehensive review, reported that appendiceal endometriosis occurs in 0.05-1.69% of women with endometriosis and up to 13.2% of those with deep infiltrating disease. When appendectomy is systematically performed in patients with advanced stages, the true prevalence may be higher than traditionally estimated. Centini et al. [7] confirmed this heterogeneity, reporting a prevalence of 2.8% in a large series of 460 patients undergoing surgery for endometriosis and noting significant associations with ovarian and bladder involvement, suggesting shared pathogenetic mechanisms such as peritoneal fluid dissemination.

The clinical manifestations of appendiceal endometriosis are diverse, ranging from incidental histological findings to acute surgical emergencies. Ross et al. [2] emphasized that diagnostic yield depends largely on histologic sampling, since small or submucosal foci may remain clinically silent. When symptomatic, appendiceal endometriosis can manifest as chronic or cyclic pelvic pain, dyschezia, hematochezia, bowel habit changes, or, less frequently, intestinal obstruction [3,7]. Mehrotra et al. [8] described a case of small bowel obstruction due to endometriosis affecting the appendix in a woman with no prior surgery, Schrempf et al. [9], in a retrospective analysis of 2,484 appendectomies, found an incidence of 0.7% of appendiceal endometriosis, most cases being incidental discoveries during evaluation for acute appendicitis, reinforcing that the condition often overlaps with gastrointestinal disorders.

Despite its varied spectrum, a frequent clinical scenario involves symptoms mimicking acute appendicitis. Drumond et al. [4], Aragone et al. [10], Bolcatto et al. [3], and Lee et al. [6] all reported cases of reproductive-age women presenting with right lower quadrant pain, leukocytosis, and radiologic findings suggestive of appendiceal inflammation, where histopathology subsequently revealed endometriosis. Imaging studies such as CT or ultrasound cannot reliably distinguish between acute appendicitis and appendiceal endometriosis [6], and preoperative suspicion remains low. Maqsoudi et al. [11] described a case of ruptured endometrioma associated with acute appendicitis, underscoring how gynecologic and gastrointestinal conditions can coexist and mimic acute surgical emergencies. This clinical overlap reinforces the importance of multidisciplinary assessment in women presenting with acute right lower quadrant pain.

Histologic examination remains the standard for diagnosis. In the present case, foci of endometrial glands and stroma were confined to the muscular layer of the appendix, without endoluminal inflammation. Similar patterns were described by Ross et al. [2], who showed that extensive histological sampling across multiple tissue levels markedly increases detection rates. Guo et al. [1] and Allahqoli et al. [5] reported variable localization of lesions, from the serosa to the muscularis, often coexisting with other pelvic implants. Kadi et al. [12] emphasized that routine histopathological assessment of all appendectomy specimens is essential, as many cases would otherwise go undiagnosed.

Laparoscopy has both diagnostic and therapeutic value in appendiceal endometriosis. It allows detailed visualization of pelvic and gastrointestinal structures, detection of associated lesions, and safe appendectomy with minimal morbidity. Guo et al. [1] and Allahqoli et al. [5] advocate for opportunistic appendectomy during endometriosis surgery, especially in advanced stages, because of its diagnostic and preventive value. Centini et al. [7] also noted that appendiceal involvement often coexists with ovarian and bladder endometriosis, reinforcing the need for systematic inspection of the appendix during laparoscopic procedures. Moreover, laparoscopy enables simultaneous treatment of suspected appendicitis and detection of incidental disease, as demonstrated in the present case, while minimizing postoperative recovery time.

When compared with previously reported cases, our patient shares several clinical and histopathological similarities but also presents notable differences. Similar to the cases of Drumond et al. [4] and Bolcatto et al. [3], the patient presented with clinical signs consistent with acute appendicitis, and the diagnosis of appendiceal endometriosis was established only after histological evaluation. However, unlike most cases where endometriotic tissue extends to the serosa or is associated with pelvic implants [1,5,7,10], our case revealed a lesion confined to the muscular layer with no other visible pelvic involvement. The absence of luminal inflammation distinguishes this presentation from cases mimicking suppurative appendicitis [6,11]. Finally, laparoscopy permitted prompt management and accurate diagnosis, consistent with the recommendations of Guo et al. [1], Centini et al. [7], and Kadi et al. [12] for comprehensive intraoperative and histopathological evaluation in women with acute abdominal pain or suspected endometriosis.

## Conclusions

Appendiceal endometriosis, although rare, represents an important diagnostic consideration in women of reproductive age presenting with acute or recurrent right lower quadrant pain. This case illustrates a distinct form of the disease, with foci of endometrial tissue confined to the muscular layer of the appendix and no evidence of luminal inflammation or pelvic implants. Such presentations emphasize the histological heterogeneity of appendiceal endometriosis and the limitations of preoperative imaging. Laparoscopy provided both diagnostic and therapeutic advantages, enabling full inspection of the pelvic cavity and histopathological confirmation. Routine evaluation of the appendix during surgery for endometriosis or acute abdominal pain may improve diagnostic accuracy and prevent missed or delayed diagnoses.
